# A case report on *Mycobacterium houstonense* infection after total hip arthroplasty

**DOI:** 10.1186/s12879-023-08705-y

**Published:** 2023-10-25

**Authors:** ZhiPeng Li, ZhaoFeng Yuan, HuiLing Cao, DaWei Huan, Yue Qiu, TianWei Xia, JiRong Shen

**Affiliations:** Affiliated Hospital of Nanjing University of Chinese Medicine, Jiangsu Provincial Hospital of Chinese Medicine, Nanjing, 210000 Jiangsu China

**Keywords:** *Rapidly growing mycobacteria*, *Mycobacteria houstonense*, Metagenomic next-generation sequencing, Periprosthetic joint Infection, Two-stage revision Surgery

## Abstract

**Background:**

*Mycobacterium houstonense* is a category of rapidly growing mycobacteria that is gram-positive, acid-fast, polycrystalline, and non-spore-forming. There have been few reports of human infection caused by *Mycobacterium houstonense* worldwide.

**Case presentation:**

We present a case of chronic periprosthetic joint infection caused by *Mycobacterium houstonense* in an elderly female patient. The patient developed signs of infection after undergoing total hip arthroplasty. Despite receiving antibiotic treatment and revision surgery, the signs of infection recurred repeatedly. Multiple bacterial cultures during the treatment period were negative. Later, we identified the pathogenic bacteria *Mycobacterium houstonense* through mNGS testing, isolated the bacteria from the ultrasonically centrifuged fluid of the prosthesis and obtained drug sensitivity results. Finally, we performed a revision surgery and treated the patient with moxifloxacin and clindamycin. After treatment, the patient did not show signs of infection recurrence during 24 months of follow-up.

**Conclusion:**

Through a relevant literature search, we believe that *Mycobacterium houstonense* may show higher sensitivity to amikacin and quinolone antibiotics. Additionally, clarifying occult infection sources through methods such as gene testing will improve the diagnosis and treatment of periprosthetic joint infection.

**Supplementary Information:**

The online version contains supplementary material available at 10.1186/s12879-023-08705-y.

## Background

Periprosthetic joint infection (PJI) is a serious complication after joint arthroplasty with a prevalence between 2.05% and 2.18% [1]. The most common pathogens are gram-positive bacteria, such as *Staphylococcus aureus* and coagulase-negative Staphylococci (60%); gram-negative bacteria can also lead to opportunistic infections [2, 3]. Among them, mycobacterial infections account for approximately 2% of all PJI cases [4]. Rare infections bring great challenges to treatment. In this case, we report a recurrent infection caused by *Mycobacterium houstonense* after total hip arthroplasty. Then, the associated literature was searched and analyzed to provide a new basis for the diagnosis and treatment of *M. houstonense*.

## Case presentation

The patient, a 75-year-old female with a past medical history of hypertension, underwent total left hip arthroplasty at another hospital in 2010. Three years later, she developed recurrent ulcers and oozing from the surgical incisions. She received treatment with antibiotics (specific pharmacological strategies unknown) and underwent surgical procedures, including drainage of abscesses, one-stage revision, and vacuum sealing drainage. Despite these interventions, her symptoms persisted, and bacterial cultures were negative during treatment.

**Fig. 1:**
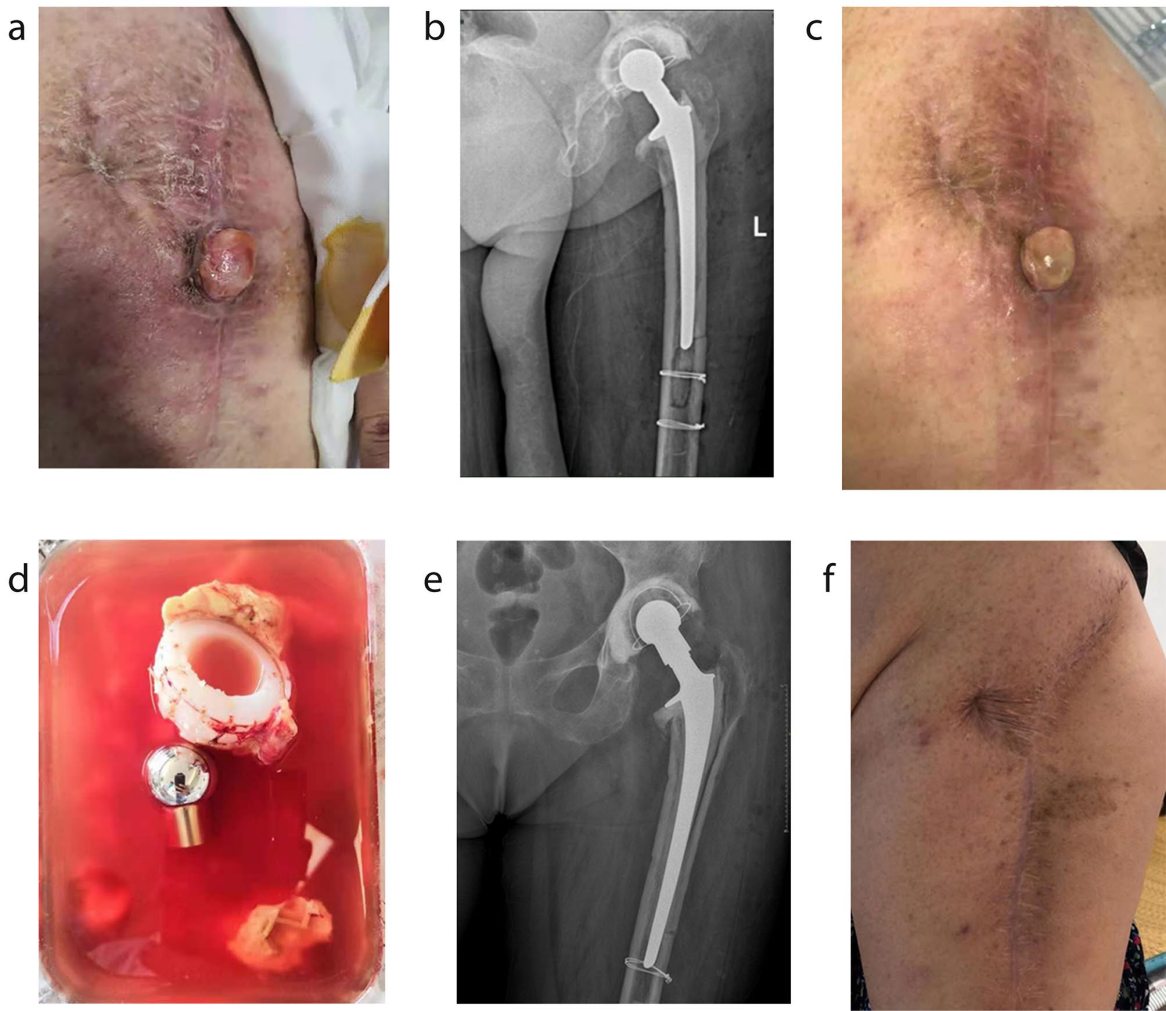
Pictures related to the treatment period. (**a**) The surgical incision at the patient’s first visit; (**b**) The X-ray image at the patient’s first visit; (**c**) The surgical incision at the patient’s second visit; (**d**) Prosthesis removed during the operation; (**e**) X-ray for the patient’s left hip joint before discharge; (**f**) The patient’s incision with 24 months’ follow-up

The patient was first seen at Jiangsu Provincial Hospital of Chinese Medicine in August 2019 (as shown in Fig. 1a, b). We performed a two-stage revision on the patient. In the first stage, we debrided the joint and removed all components. We then replaced the left hip with vancomycin bone cement. After the first surgery, the patient received vancomycin intravenously for two weeks and took oral levofloxacin and rifampicin for three months for infection control. Three months later, the patient’s incision had healed well, and two repeat blood and CRP tests showed no abnormalities. We therefore proceeded with a second-stage revision. During the operation, we observed slight inflammatory synovial tissue hyperplasia in the joint cavity. Although the incision had a good postoperative recovery, the patient continued taking oral levofloxacin and rifampicin for three months to prevent infection. Bacterial cultures taken during treatment were negative.

In May 2020, the patient returned to our hospital due to yellowish cloudy secretions exuding from the incision of the hip, accompanied by superficial proliferation of granulation tissue (as shown in Fig. 1c). Given the history of recurrent incisional infections, we decided to perform a one-stage revision, which involved the removal of all components and cement and replacement with a bone cement prosthesis. Following surgery, we sent tissue from the sinus tract to BGI Genomics (Shenzhen, China) for metagenomic next-generation sequencing (mNGS) testing. The test revealed an infection with *M. houstonense*. Due to the inability to obtain drug susceptibility test results from cultures of the joint fluid, we prescribed a combination of clarithromycin and cefoxitin orally for three months based on the relevant literature to treat the infection [5].

Subsequently, the patient experienced intermittent infections, prompting us to perform several retention-prosthesis debridement procedures while performing bacterial cultures with infected tissues. The bacterial cultures revealed a variety of highly drug-resistant bacteria, including *Staphylococcus capitis*, *Staphylococcus haemolyticus*, and *Staphylococcus epidermidis*. Based on the susceptibility testing results, we had modified the anti-infective regimen to include intravenous drip meropenem for one week, followed by oral therapy consisting of rifampicin, clarithromycin, and cefoxitin.

In November 2020, the patient’s incision exhibited clinical signs of infection again, with yellowish cloudy secretions. After consultation, we discontinued the antibiotics for two weeks and performed a prosthetic debridement and revision surgery. During the intraoperative exploration, the femoral stem did not show any loosening, so we retained the femoral stalk prosthesis and only removed the acetabulum and ball-head prosthesis (as shown in Fig. 1d) and placed a cemented acetabular cup after thorough debridement.

We placed the removed joint prosthesis and wash fluid in a sterile environment and processed the joint prosthesis specimen by vortexing for 30 s, ultrasound at 40 Hz for 5 min, and repeating vortexing for 30 s to obtain the wash fluid. A portion of the wash fluid was sent for mNGS testing, and the mNGS results indicated infection by *Mycobacterium houstonense* (The test reports were shown in supplementary materials). Then, we injected 10 ml of wash fluid into blood culture bottles (one bottle for aerobes and one bottle for anaerobes). Additionally, we took a portion of the wash fluid and centrifuged it at 3500 rpm for 5 min. The resulting precipitates were inoculated on Columbia blood agar medium. After 72 h of culture, small dry colonies were grown; after continuation for 24 h, the cultures showed dry and wrinkled colonies. Automated mass spectrometry (MODI-TOF MS) identified them as *Mycobacterium fortuitum*. In addition, the aerobic bottle became positive after 102.7 h, and pathogenic bacteria were subcultured and identified as *M. fortuitum*, consistent with the mNGS test result (mNGS only identified *M. houstonense*, which belongs to the group of *M. fortuitum*). A broth microdilution method was used to determine the minimal inhibitory concentration (MIC) according to CLSI M24 A2 [6], and the drug susceptibility testing results showed that *M. houstonense* was sensitive to amikacin and moxifloxacin (as shown in Table 1).

**Table 1:** Antimicrobial sensitivity of *Mycobacterium houstonense*

Antibacterial drugs	Breakpoints (µg/ml)			*Mycobacterium houstonense*	
	Susceptible	Intermediate	Resistant	MIC (µg/ml)	Results
Moxifloxacin	≤ 1	2	≥ 4	0.25	sensitive
Amikacin	≤ 16	32	≥ 64	8	sensitive
Clarithromycin	≤ 2	4	≥ 8	256	resistant
Levofloxacin	—	—	—	16	—
Rifampin	—	—	—	≥ 64	—
Ethambutol	—	—	—	256	—
Isoniazid	—	—	—	64	—
Cefoxitin	≤ 16	32–64	≥ 128	/	/
Ciprofloxacin	≤ 1	2	≥ 4	/	/
Doxycycline	≤ 1	2–4	≥ 8	/	/
Imipenem	≤ 4	8–16	≥ 32	/	/
Linezolid	≤ 8	16	≥ 32	/	/
Meropenem	≤ 4	8–16	≥ 32	/	/
Trimethoprim-sulfamethoxazole	≤ 2/38	—	≥ 4/76	/	/
Tobramycin	≤ 2	4	≥ 8	/	/

Note: —: No Breakpoint in CLSI M24-A2;

/: Recommended drugs in CLSI M24-A2 which were not tested due to the limitations of the detection facilities and clinical medication situations

Based on the sensitivities and clinical experience, we replaced the antibiotics with oral moxifloxacin and clindamycin for three months. Thereafter, no abnormalities in routine blood or CRP results were observed. The patient was followed for 24 months without signs of any infection (as shown in Fig. 1e, f).

## Discussion

This is the first reported case of periprosthetic joint infection caused by *Mycobacterium houstonense* after total hip arthroplasty.

### Mycobacterium houstonense

*Mycobacterium houstonense* was first isolated from a patient with a facial infection in Houston, USA, in 2004 and was named after the city [7]. Based on its growth rate, *Mycobacterium houstonense* belongs to the *Mycobacterium fortuitum* clade, which is a group of rapidly growing mycobacteria (RGM) within the nontuberculous mycobacteria (NTM) group. It is an irregularly shaped, gram-positive bacillus that cannot move by itself, is usually acid-fast, and does not form aerial mycelium or spores. The growth characteristics of *Mycobacterium houstonense* are not well known, but NTMs are typically found in soil and natural water sources [8, 9]. Although it is difficult to trace the source of infection, it is generally believed that human RGM infection is acquired from the environment, and there is no evidence of person-to-person spread of NTM [10–12]. The techniques for identifying NTM mainly involve commercial DNA probes and high-performance liquid chromatography (HPLC). However, in some cases especially infection for RGM, extended antibiotic in vitro susceptibility testing, DNA sequencing, or polymerase chain reaction (PCR) restriction endonuclease assay (PRA) may also be necessary [5]. Therefore, genetic testing such as NGS or mNGS is crucial for identifying pathogenic microorganisms in cases of RGM infection. Infections with *Mycobacterium houstonense* are extremely rare, with only the following three reported cases in the literature: ocular [13], intracranial [14], and infection of an open humerus fracture [15] (as shown in Table 2). All patients had a history of immunodeficiency or exposure to water or soil.

**Table 2:** Basic information of 4 patients

Case	1 [13]	2 [14]	3 [15]	4 (this case)
Age	45	26	68	77
Gender	male	male	male	female
Primary disease	AGV implant surgery	meningitis	Postoperative of open humerus fracture	total-hip arthroplasty
Comorbidity	Non	Non	—	Hypertension
Interval time	3 years	—	3 weeks	3 years
Suspected source of infection	aquaculture	immunodeficiency、geographical factors	water, soil or dust	soil or dust
Testing method	16 S rRNA	mNGS	16 S rRNA	mNGS
Sensitive antibiotic	levofloxacin, ciprofloxacin, amikacin	—	levofloxacin, moxifloxacin, amikacin	moxifloxacin, amikacin
Resistant antibiotic	doxycycline, sulfamethoxazole, tobramycin	—	cefoxitin, doxycycline, linezolid, imipenem, tobramycin, clarithromycin, trimethoprim sulfamethoxazole	clarithromycin
Treatment regimen	intravenous amikacin and oral levofloxacin	amikacin, tigecycline, clarithromycin and imipenem(meropenem)	Intravenous levofloxacin and injection of amikacin	oral moxifloxacin and clindamycin
Ending	cure	death	cure	cure
Follow-up time	4 months	—	not followed up	24 months

In this case, the patient was a farmer, and we suspect *Mycobacterium houstonense* infection was due to improper postoperative care after the patient’s initial total hip replacement and exposure to the soil environment. Mycobacterium are immovable, resulting in bacterial colonization and biofilm formation on the surface of the prosthesis [16]. The biofilm matrix is typically composed of extracellular polysaccharides, proteins, and extracellular DNA, which provide microbes with tolerance to antibiotics and host immune clearance [17]. Currently, tissue bacterial culture is the most common test to used identify the source of infection. However, traditional bacterial culture testing is time-consuming and has a low detection rate of less than 50% [18]. To improve the culture positivity rate, Schfer et al. [19] suggested extending the microbial culture time for more than two weeks, while Peel et al. [20] proposed inoculating three periprosthetic tissue specimens into blood culture bottles or culturing four specimens in standard plates and broth cultures. For biofilms, sonicated prostheses can help peel off the biofilm to improve the detection rate [21].

Genetic testing is currently the most sensitive technology for pathogen detection. mNGS is based on a shotgun method and Sanger sequencing, which directly detect all nucleic acids from the sample, theoretically detecting all pathogens [22, 23]. Thoendel et al. [24] found that mNGS could identify pathogens in positive cultures of PJI with high probability (94.8%) and detect other potential pathogens (9.6%); mNGS could even detect new potential pathogens in 43.9% of negative culture results. It has also been suggested that mNGS is sensitive to low levels of microorganisms or residual nucleic acids in the sample, so antibiotics have less impact on its results [25].

In addition, with the increase in newly identified species, it is essential to use molecular-level technology to make a definitive diagnosis at the species level, which contributes to selecting a more targeted treatment plan [26]. In this case, the same set of wash fluid was identified as *Mycobacterium fortuitum* in laboratory bacterial culture, while mNGS more precisely identified the species as *Mycobacterium houstonense*. Therefore, the mNGS assay is helpful for early pathogen identification and culture [27].

In addition to this case, we reviewed the available case reports of *Mycobacterium houstonense.* The cases have shown sensitivity to amikacin and quinolone antibiotics in *Mycobacterium houstonense* but resistance to clarithromycin. There are currently no recommended medications for treating *Mycobacterium houstonense* in clinical guidelines. The American Thoracic Society (ATS) recommends using drugs for occasional mycobacteria based on their drug sensitivity results only [5]. Therefore, in cases where *Mycobacterium houstonense* infection is confirmed but drug sensitivity results are not available, we recommend attempting treatment with amikacin or quinolone antibiotics.

### Periprosthetic joint Infection after total hip arthroplasty

With the increasing prevalence of hip arthroplasty, the incidence of PJI is projected to rise to 6.5% by 2030 [28]. Moreover, infections caused by rare bacteria with low toxicity are on the rise, and biofilm formation on the prosthetic surface may be one of the most significant factors contributing to recurrent infections, delayed healing, and negative bacterial cultures in PJI patients. In this case, although the patient’s symptoms improved in stages with antibiotic treatment, signs of infection recurred due to the failure to identify the causative organism and its sensitivity to antibiotics. Therefore, timely identification of the causative organism and its corresponding sensitive antibiotics are critical for the effective management of PJI patients.

Revision surgery is currently the most common treatment for PJI, particularly for complex cases. It is also the ultimate surgical treatment for PJI. Two-stage revision is considered the most effective surgical technique for treating chronic PJI, with a postoperative infection-free survival rate of 80-100% [29]. Theoretically, the source of infection needs to be completely controlled before second-stage revision. Related studies have shown that a positive culture at the second-stage revision is independently associated with twice the risk of subsequent failure [30]. However, there are currently no uniform criteria for the timing of prosthetic reimplantation and indicators of infection control [31]. Clinical assessment, regression of inflammatory indicators, and negative joint aspiration are factors to be considered before a second-stage surgery [32]. The synovial leukocyte count has also been shown to be an accurate indicator for infection control [33]. During the first-stage surgery, we recommend the use of ultrasonic shock centrifugation for the removed prosthesis, with the centrifuge fluid sent for mNGS testing to increase the detection rate for occult pathogens. Additionally, mNGS testing should be repeated prior to the second-stage surgery to ensure that the infection is fully controlled at the time of prosthesis reimplantation, thus reducing the rate of failure of two-stage revision.

Overall, the early identification of the causative organism and selection of appropriate surgical procedures are two essential conditions for effective treatment of the disease.

## Conclusion

For infections caused by *Mycobacterium houstonense*, amikacin or quinolone antibiotics may be used to achieve better treatment effects. For rare bacterial infections, it is crucial to identify the infection source as early as possible. At the same time, mNGS identification of the pathogen at the species level is beneficial for developing more accurate treatment plans.

### Electronic supplementary material

Below is the link to the electronic supplementary material.

Supplementary Material 1

Supplementary Material 2

Supplementary Material 3

Supplementary Material 4

Supplementary Material 5

## Data Availability

The raw data supporting the conclusions of this article will be made available by the authors, without undue reservation.

## Abbreviations

RGM: Rapidly growing mycobacteria
PJI: Periprosthetic joint infection
mNGS: Metagenomic next-generation sequencing
NTM: Non-tuberculous Mycobacteria
