# Supplementary material for: A case report on Mycobacterium houstonense infection after total hip arthroplasty
Source: BMC Infect Dis. 2023 Oct 25;23:722. doi: 10.1186/s12879-023-08705-y (PMC10598912; doi:10.1186/s12879-023-08705-y)
Supplement: Supplementary file 1 — Supplementary Material 1 [file 12879_2023_8705_MOESM1_ESM.docx]

|  | DATE | WBC | NE% | CRP |
| --- | --- | --- | --- | --- |
| First admission（2019） | 8.29 | 4.1 | 58 | 13.5 |
|  | 9.5 | 16.86 | 92.6 |  |
|  | 9.9 | 6 | 62 | 13 |
|  | 9.16 | 6.5 | 63.9 | 69 |
|  | 9.23 | 4.2 | 62.7 | 51.5 |
| Second admission（2019） | 11.26 | 4.7 | 61.4 | 10.2 |
|  | 12.02 | 4.1 | 55.1 | 12.6 |
|  | 12.06 | 11.8 | 84 | 124 |
|  | 12.12 | 3.9 | 58.5 | 13 |
|  | 12.18 | 4.2 | 53.8 | 7.19 |
| Third admission（2020-2021） | 5.9 | 4.6 | 58 | 10.7 |
|  | 5.14 | 9.15 | 77.5 |  |
|  | 5.16 | 4.8 | 67.2 | 91.4 |
|  | 5.19 | 4.6 | 69.8 | 26 |
|  | 5.23 | 4.9 | 77.1 | 7.52 |
|  | 5.26 | 4.3 | 62 | 15.3 |
|  | 6.08 | 4.06 | 61.8 | 41.5 |
|  | 6.18 | 4.8 | 62.8 | 36.8 |
|  | 7.24 | 3.9 | 85.3 | 40.6 |
|  | 7.27 | 4.37 | 57 | 11.4 |
|  | 8.13 | 5.06 | 59.7 |  |
|  | 8.15 | 4.28 | 86 |  |
|  | 8.20 | 4.1 | 60.4 | 26.1 |
|  | 9.1 | 3.56 | 60.4 | 31.5 |
|  | 9.7 | 4.45 | 62.3 | 36.2 |
|  | 9.11 | 4.04 | 60.1 | 26.8 |
|  | 9.15 | 5.84 | 59 | 18.5 |
|  | 9.19 | 4.14 | 55.8 | 19.9 |
|  | 9.23 | 4.03 | 53.7 | 17.9 |
|  | 9.29 | 3.39 | 58.9 | 18.8 |
|  | 10.5 | 3.74 | 58.6 | 19.7 |
|  | 10.10 | 3.49 | 58.6 | 15.6 |
|  | 10.15 | 3.68 | 51.7 | 20.5 |
|  | 10.20 | 4.01 | 60.6 | 15.5 |
|  | 10.26 | 3.98 | 56 | 15.9 |
|  | 11.2 | 3.56 | 55.6 | 15.8 |
|  | 11.5 | 3.8 | 62.4 | 13.6 |
|  | 11.10 | 3.97 | 60.8 | 15.8 |
|  | 11.14 | 3.41 | 61 | 14.1 |
|  | 11.20 | 3.91 | 56.5 | 7.87 |
|  | 11.27 | 3.35 | 55.7 | 8.43 |
|  | 12.01 | 5.17 | 66.3 | 43.9 |
|  | 12.10 | 7.15 | 82.2 | 75.6 |
|  | 12.14 | 3.8 | 63.3 | 7.54 |
|  | 12.18 | 3.92 | 63.3 | 5.89 |
|  | 12.24 | 3.44 | 61 | 2.59 |
|  | 12.31 | 3 | 55.7 | 2.74 |
|  | 1.12 | 3.3 | 61.2 | 3.83 |
|  | 1.14 | 3.31 | 61.6 | 4.55 |
|  | 1.20 | 3.5 | 58.6 | 4.39 |
