# Supplementary material for: A case report on Mycobacterium houstonense infection after total hip arthroplasty
Source: BMC Infect Dis. 2023 Oct 25;23:722. doi: 10.1186/s12879-023-08705-y (PMC10598912; doi:10.1186/s12879-023-08705-y)
Supplement: Supplementary file 4 — Supplementary Material 4 [file 12879_2023_8705_MOESM4_ESM.docx]

**Report Card for High-Throughput Genetic Testing of Pathogenic Microorganisms**

Name: Ms. Ji Sampling date: 2020-12-09 Sample number: 20S4668140

**sequencing results**

**1. Bacteria**

| Typology | Genus | | Species | |
| --- | --- | --- | --- | --- |
|  | Latin name | Number of sequences detected^#^ | Latin name | Number of sequences detected^#^ |
| G^+^ | Mycolicibacter | 16 | Mycolicibacterium houstonense | 8 |

(1)typology：G^+^ (Gram-positive bacterium)/G- (Gram-negative bacterium)

(2)Number of sequences detected#: Number of rigorously compared sequences of this microorganism detected at genus/species level

**2. Fungi**

| Genus | | Species | |
| --- | --- | --- | --- |
| Latin name | Number of sequences detected^#^ | Latin name | Number of sequences detected^#^ |
| Not found | | | |

**3. Virus**

| Typology | Species | | Subtype | |
| --- | --- | --- | --- | --- |
|  | Latin name | Number of sequences detected^#^ | Latin name | Number of sequences detected^#^ |
| Not found | | | | |

**4. parasite**

| Genus | | Species | |
| --- | --- | --- | --- |
| Latin name | Number of sequences detected^#^ | Latin name | Number of sequences detected^#^ |
| Not found | | | |

**5. Mycobacterium tuberculosis complex**

| Species complex | | Species | |
| --- | --- | --- | --- |
| Latin name | Number of sequences detected^#^ | Latin name | Number of sequences detected^#^ |
| Not found | | | |

**6. Mycoplasma/chlamydia/rickettsia**

| Genus | | Species | |
| --- | --- | --- | --- |
| Latin name | Number of sequences detected^#^ | Latin name | Number of sequences detected^#^ |
| Not found | | | |
