# Supplementary figures and images for: A case report on Mycobacterium houstonense infection after total hip arthroplasty
Source: BMC Infect Dis. 2023 Oct 25;23:722. doi: 10.1186/s12879-023-08705-y (PMC10598912; doi:10.1186/s12879-023-08705-y)

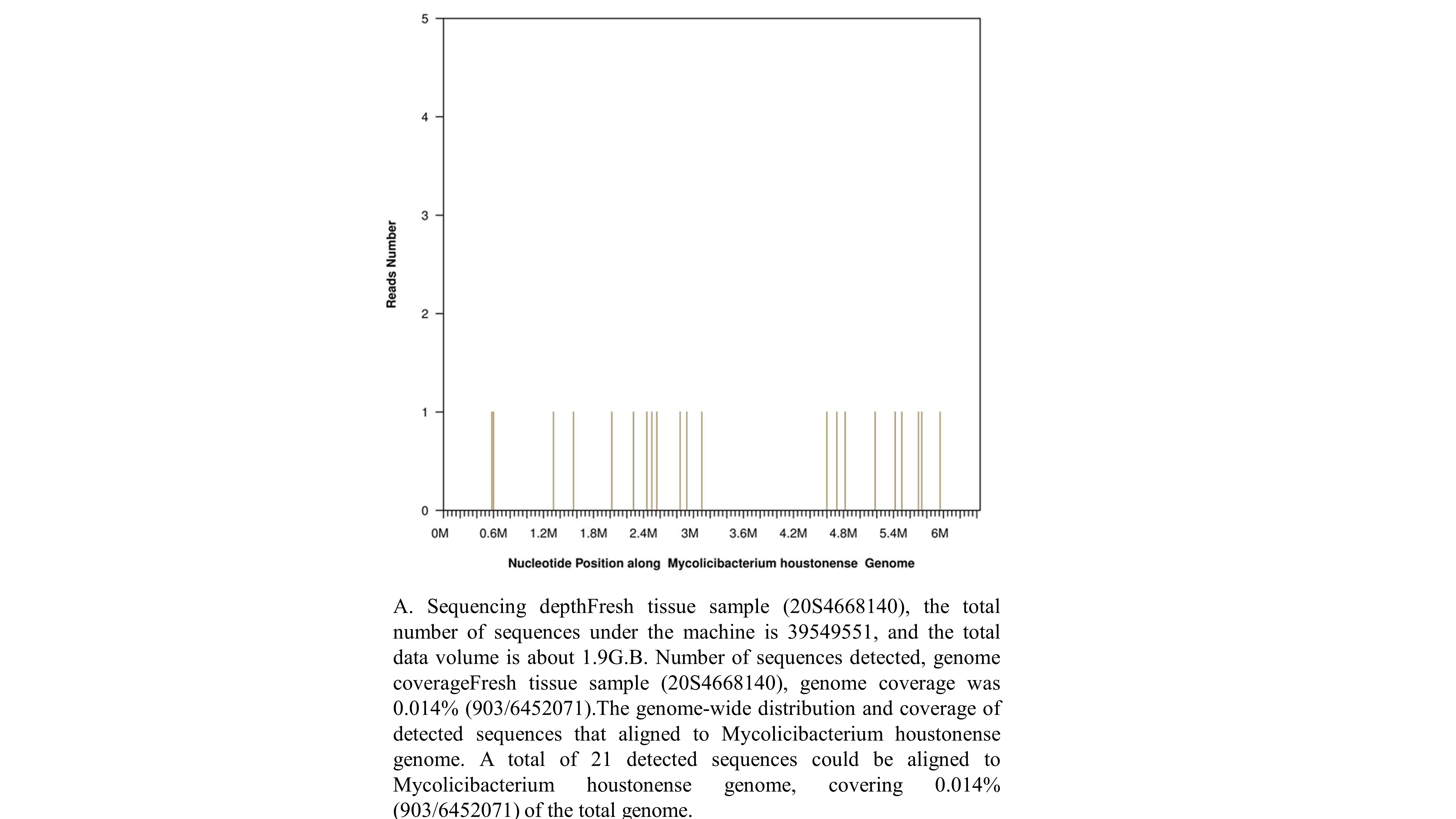

Supplement: Supplementary file 5 — Supplementary Material 5 [file 12879_2023_8705_MOESM5_ESM.png]
